# Efficacy and safety of dupilumab for the treatment of uncontrolled asthma: a meta-analysis of randomized clinical trials

**DOI:** 10.1186/s12931-019-1065-3

**Published:** 2019-05-31

**Authors:** Xiao-feng Xiong, Min Zhu, Hong-xia Wu, Li-li Fan, De-yun Cheng

**Affiliations:** Department of Respiratory and Critical Care Medicine, West China Hospital, Sichuan University, NO. 37 Guoxue Alley, Chengdu, 610041 Sichuan China

**Keywords:** Dupilumab, Meta-analysis, Randomized control trial, Uncontrolled asthma

## Abstract

**Background:**

Several recent clinical trials have assessed the effects of dupilumab in uncontrolled asthma, but reached no definite conclusion. We therefore conducted this meta-analysis to evaluate the overall efficacy and safety of dupilumab for the treatment of uncontrolled asthma.

**Methods:**

All randomized controlled trials were included. Standard mean differences (SMD) or relative risks (RR) were calculated using Fixed-or random-effects models.

**Results:**

Five studies involving 3369 patients were identified. Pooled analysis showed significant improvements in the first-second forced expiratory volume (FEV_1_) (SMD = 4.29, 95% CI: 2.78–5.81) and Asthma Quality of Life Questionnaire scores (SMD = 4.39, 95% CI: 1.44–7.34). Dupilumab treatments were also associated with significantly decreased 5-item Asthma Control Questionnaire scores (SMD = − 4.95, 95% CI: − 7.30 to − 2.60), AM and PM asthma symptom scores (SMD = − 5.09, 95% CI: − 6.40 to − 3.77; SMD = − 4.92, 95% CI: − 5.98 to − 3.86, respectively), and severe exacerbation risk (RR = 0.73; 95% CI: 0.67–0.79) compared with placebo, with similar incidence of adverse events (RR = 1.0; 95% CI: 0.96–1.04).

**Conclusion:**

Dupilumab treatment is relatively well-tolerated and could significantly improve FEV_1_, symptoms, asthma control, and quality of life, and reduced severe exacerbation risk in patients with uncontrolled asthma.

## Introduction

Asthma is one of the most common chronic diseases and affects around 334 million people worldwide [1], of whom approximately 20–25% have uncontrolled disease [2]. Many of these patients have substantially reduced lung function and a higher risk of disease exacerbation, despite maximum treatment, and require the use of a considerable proportion of health care resources [3, 4]. Immunologically, type 2 cytokines (specifically interleukin [IL]-4, IL-5, and IL-13) are recognized as playing a substantial pathobiological role in asthma [5–7]. These cytokines link to a type-2/T-helper-2-cell (Th2)-high molecular asthma phenotype in up to 50% of asthmatic patients, across all levels of severity [8–10].

Dupilumab, a fully human monoclonal antibody, is directed against the alpha subunit of the IL-4 receptor, thereby blocking both IL-4 and IL-13 signaling, and hence type 2 inflammation [11]. It has been approved for the treatment of moderate-to-severe atopic dermatitis [12]. Several randomized, placebo-controlled studies (RCTs) have shown efficacy of dupilumab in patients with uncontrolled asthma. However, because the sample sizes of these studies were varied and results were less consistent, the evidence is insufficient for drawing robust conclusions. Therefore, we conducted a meta-analysis to assess the overall efficacy and safety of dupilumab treatment for uncontrolled asthma.

## Methods

### Data sources and searches

We searched PubMed, Embase, the Cochrane Library and Chinese Biological Medicine (CBM) databases for articles published up to June 30, 2018, to identify all trials assessing dupilumab therapy for patients with uncontrolled asthma, using the search terms: “asthma” and “dupilumab”. Publication species was limited to humans. In addition, relevant review articles and their reference lists were checked manually.

### Study selection

Studies were considered eligible if they met the following criteria: 1) trials recruited adults/adolescents (≥ 12 years old) diagnosed with uncontrolled asthma, 2) participants received dupilumab therapy at any dose, 3) RCTs, and 4) RCTs reporting the following outcomes: lung function (FEV_1_), he 5-item Asthma Control Questionnaire (ACQ-5) score, fractional exhaled nitric oxide (FE_NO_), AM and PM asthma symptom scores, quality of life (AQLQ), severe exacerbation rate, or adverse events. Two investigators (XFX and MZ) independently screened all references according to the selection criteria. Disagreements were resolved through discussion.

The patients were diagnosed with asthma persisting for 1 year or more, according to the Global Initiative for Asthma 2009 or 2014 guidelines [13, 14]. Uncontrolled asthma was defined based on current treatment with a medium-to-high-dose inhaled glucocorticoid (fluticasone propionate at a total daily dose of ≥500 μg or equipotent equivalent), plus up to 2 additional controllers (e.g., a long-acting β2-agonist or leukotriene receptor antagonist); a forced expiratory volume in 1 s (FEV_1_) ≤ 80% of the predicted normal value before bronchodilator use (or ≤ 90% of the predicted normal value in those 12–17 years of age); FEV_1_ reversibility of at least 12% and 200 ml [15]; the 5-item Asthma Control Questionnaire (ACQ-5) score ≥ 1.5 (on a scale from 0 [no impairment] to 6 [maximum impairment]; the minimal clinically important difference is 0.5) [16]; and a worsening of asthma in the previous year that led to hospitalization, emergency medical care, or treatment with systemic glucocorticoids ≥3 days [15]. Asthma symptom scores are patient-reported measures of asthma symptoms, taken upon waking and in the evening. Their effects on activities (PM) and sleep (AM) were noted. These symptom scores range from 0 to 4, with higher scores indicating greater disruption [15]. A severe exacerbation was defined as a deterioration of asthma that required the use of systemic corticosteroids for at least 3 days, or hospital admission, or an emergency department visit because of asthma, treated with systemic corticosteroids [17]. The AQLQ is a patient-reported measure of the effect of asthma on quality of life; higher scores indicate a better quality of life; a global score is calculated and ranges from 0 to 7 [15].

### Data extraction and quality assessment

The Preferred Reporting Items for Systemic Reviews and Meta-Analyses (PRISMA) statement was followed. Two authors (XFX and MZ) performed data extracted and recorded desirable information of each enrolled study in a standard form as recommended by Cochrane [18]. Any disagreement was resolved through discussion or adjudicated by a third author (HXW). In addition, we evaluated the risk of bias using the Cochrane Collaboration’s domains [19].

### Statistical analyses

Intervention effects were presented using risk ratios (RR) and 95% confidence intervals (CIs) for dichotomous data and using mean differences (MD) and 95% CIs for continuous data. If a trial presented more than 2 intervention groups, we combined 2 or 3 groups into a single group according to the Cochrane handbook [19]. Heterogeneity was quantified by I^2^ statistic and the χ^2^ test. Random-effects model was performed in the presence of high heterogeneity (I^2^ > 50%); otherwise, fixed-effects model was applied [20]. Meta-regression was used to explore potential sources of heterogeneity. Funnel plots with the Begg’s and Egger’s tests were used to test publication bias [21]. All statistical analysis was accomplished by an independent statistician using Review Manager (Version 5.3, The Cochrane Collaboration, Copenhagen) and Stata (Version 12.0, Stata Corporation, USA), and rendered statistical significance as *P*-value < 0·05.

Meta-analyses may cause type I errors because of sparse data and repetitive testing of accumulating data [22]. We performed trial sequential analysis (TSA) to assess the risk of type I errors, which can identify whether the evidence in a meta-analysis is reliable and authentic. If the cumulative z curve crosses the trial, the boundaries, and the required information size, the evidence is sufficient to reach a conclusion, and no further studies are needed. We calculated the required information size for FEV_1_ using α = 0.05 (2-sided) and β = 0.20 (power of 80%). TSA version 0.9 beta (http://www.ctu.dk/tsa) was applied for the analyses [23].

## Results

### Study identification

**Fig. 1:**
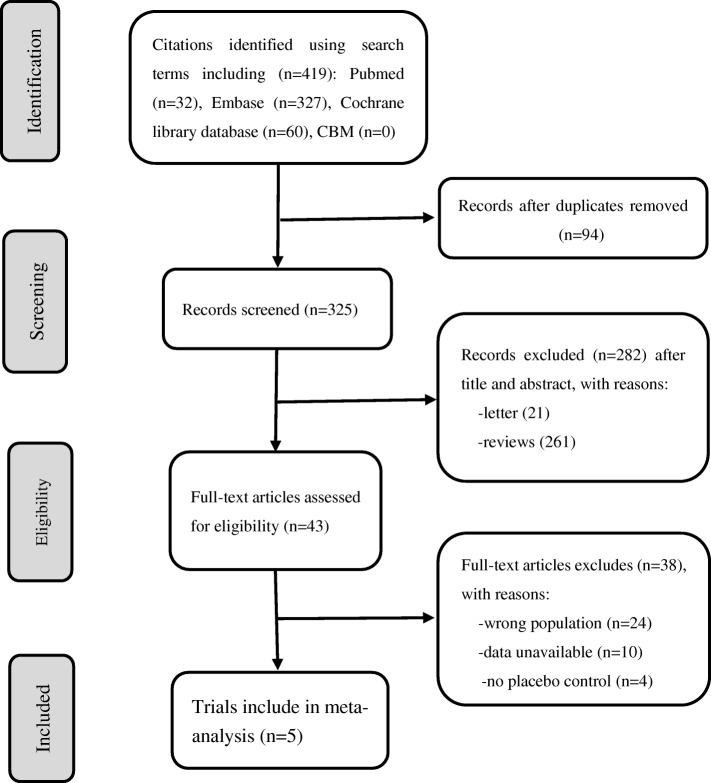
Flow chart of study identification, inclusion, and exclusion

The initial database search resulted in 419 articles. After reviewing the titles and abstracts, 43 were found to be relevant for further detailed evaluation. Of these 38 were excluded as the population was wrong (*n* = 24), there was no placebo control (*n* = 4), or data were unavailable (*n* = 10). Thus, 5 RCTs were included in the meta-analysis [15, 24–27] (Fig. 1).

### Study characteristics

We enrolled 5 studies with 3369 patients (Table 1). The sample sizes ranged from 52 to 632 subjects. A single intervention group (dupilumab 300 mg qw and 300 mg q2w) was presented in 2 trials, and the remaining studies included 2 or more interventions (dupilumab 200 mg q2w, 200 mg q4w, 300 mg q2w, 300 mg q4w). Outcome reporting varied among the trials. FEV_1_ was reported in 5 studies [15, 24–27]. Severe asthma exacerbations rate was reported in 4 trials [15, 24–26]. ACQ-5 scores, FE_NO_, and AM and PM asthma symptom scores were reported in 3 trials [15, 26, 27]. AQLQ was reported in 2 trials [15, 26].

**Table 1:** Characteristic of randomized controlled trials included

Reference	phase	Country	Asthma severity	Patients(n) dupilumab/placebo	Treatment duration	Follow up	Dosage	Outcomes
Wenzel 2013 [27]	2A	The US	Moderate-to-severe; blood eosinophil count of at least 300 cells/μl	52/52	12 weeks	8 weeks	300 mg q1w	FEV_1_; PEF; ACQ5 score; FE_NO_; Asthma exacerbations; Morning and evening asthma score
Wenzel 2016 [15]	2b	Multinational	Uncontrolled moderate-to-severe asthma	611/158	24 weeks	12 weeks	200 mg q4w 300 mg q4w 200 mg q2w 300 mg q2w	FEV_1_; ACQ5 score; FE_NO_; AQLQ; Severe asthma exacerbations; Morning and evening asthma score
Castro 2018 [26]	3	Multinational	Uncontrolled moderate-to-severe asthma	Group 1: 631/313 Group 2: 632/321	52 weeks	12 weeks	200 mg q2w 300 mg q2w	FEV_1_; PEF; ACQ5 score; FE_NO_; AQLQ; Severe asthma exacerba-tions; Morning and evening asthma score
Rabe 2018 [25]	3	Multinational	Oral glucocorticoid–dependent severe asthma	103/107	24 weeks	NM	300 mg q2w	FEV_1_; FE_NO_; Severe asthma exacerbations Reduction in the oral glucocorticoid dose
Weinstein 2018 [24]	2b	Multinational	Uncontrolled persistent asthma	Group 1: 92/56 Group 2: 157/84	24 weeks	NM	200 mg q2w 300 mg q2w	FEV_1_; Severe asthma exacerbations

*Abbreviations*: *FEV*_*1*_ forced expiratory volume in 1 s, *PEF* peak expiratory flow, *ACQ-5* 5-item Asthma Control Questionnaire, *FE*_*NO*_ fractional exhaled nitric oxide, *AQLQ* the Asthma Quality of Life Questionnaire, *NM* not mentioned, *q1w* once weekly, *q2w* every 2 weeks, *q4w* every 4 weeks

### Trial sequential analysis

**Fig. 2:**
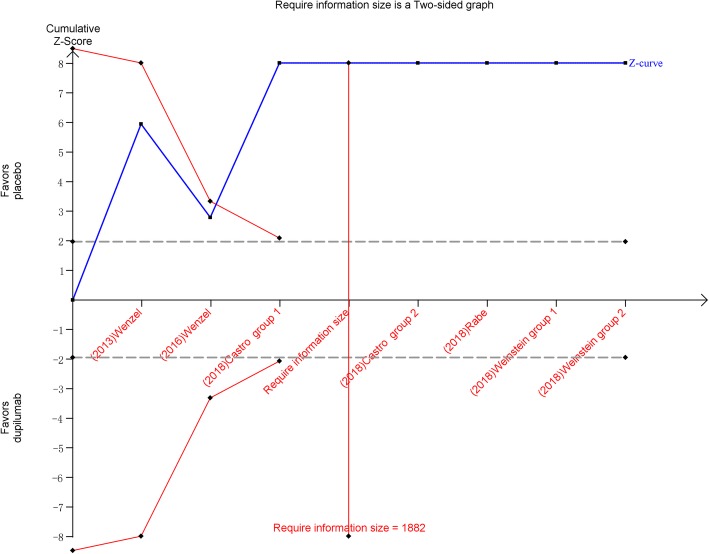
Trial sequential analysis of 5 trials comparing dupilumab with placebo for FEV_1_. Trial sequential analysis of 7 groups (two trial contains two groups) illustrating that the cumulative z curve crossed both the conventional boundary and the trial sequential monitoring boundary, establishing sufficient and conclusive evidence and suggesting that further trials are not required. Using α = 0.05 (two-sided) and β = 0.20 (power of 80%) calculate that the optimal sample size was 1882 patients

TSA found that the optimal sample size required for reliable detection of a reasonable effect of dupilumab treatment on FEV_1_ of asthma was 1882 participants; the number of patients included in our study far exceeded this number. TSA presented that the cumulative z curve crossed both the conventional boundary and the sequential monitoring boundary, which indicated that the cumulative evidence is reliable and authentic. Therefore, further studies were not needed (Fig. 2).

### Efficacy

**Fig. 3:**
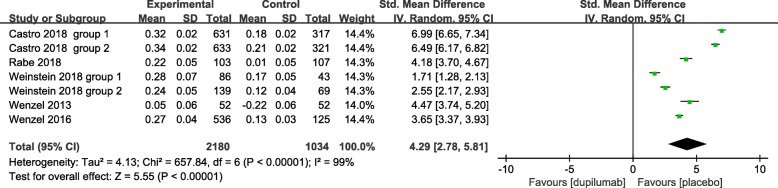
The effect of dupilumab versus placebo on FEV_1_. SD = standard derivation, IV = Inverse Variance, CI = confidence interval, Std. Mean Difference = standardized mean difference

**Fig. 4:**
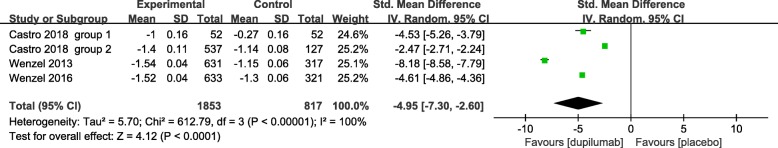
The effect of dupilumab versus placebo on ACQ-5 score. ACQ-5 = 5-item Asthma Control Questionnaire, SD = standard derivation, IV = Inverse Variance, CI = confidence interval, Std. Mean Difference = standardized mean difference

**Fig. 5:**
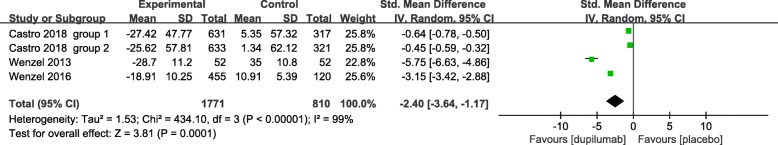
The effect of dupilumab versus placebo on FE_NO._ FE_NO =_ fractional exhaled nitric oxide SD = standard derivation, IV = Inverse Variance, CI = confidence interval, Std. Mean Difference = standardized mean difference

**Fig. 6:**
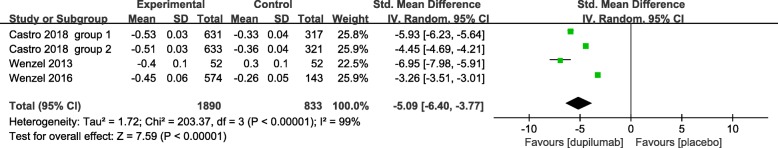
The effect of dupilumab versus placebo on AM asthma symptom score

**Fig. 7:**
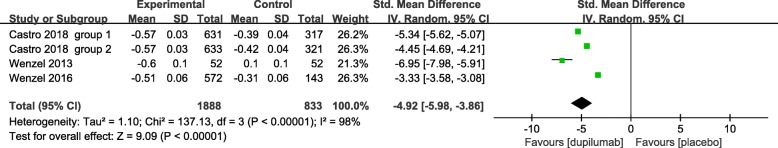
The effect of dupilumab versus placebo on PM asthma symptom score. SD = standard derivation, IV = Inverse Variance, CI = confidence interval, Std. Mean Difference = standardized mean difference

**Fig. 8:**
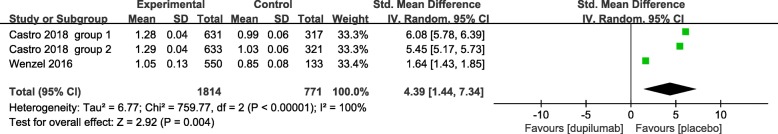
The effect of dupilumab versus placebo on AQLQ. AQLQ = Asthma Quality of Life Questionnaire, SD = standard derivation, IV = Inverse Variance, CI = confidence interval, Std. Mean Difference = standardized mean difference

**Fig. 9:**
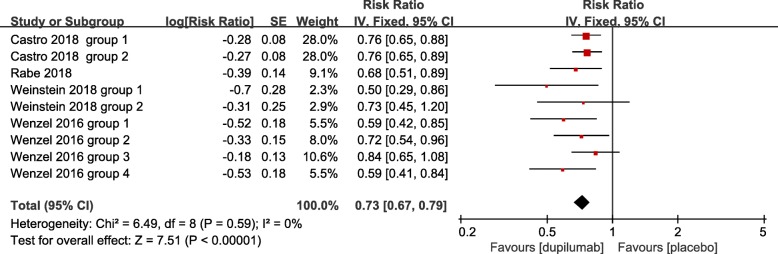
Forest plot of the effect of dupilumab treatment on asthma severe exacerbations verse placebo. Fixed-effects model. RR = relative risk, CI = confidence interval

We assessed severity measures for efficacy including lung function (FEV_1_), ACQ-5 score, FE_NO_, AM and PM asthma symptom scores, severe asthma exacerbation rate, and quality of life (AQLQ). Overall, our studies illustrated significant improvement in the efficacy in the treatment of asthma with the use of dupilumab in terms of all clinical indexes. The pooled analyses indicated that dupilumab treatment significantly improved FEV_1_ (SMD = 4.29, 95% CI: 2.78 to 5.81, *P* < 0.001). However, there was a high level of heterogeneity (I^2^ = 99%, *P* < 0.001) (Fig. 3), with no evidence of publication bias (Egger’s test *P* = 1.00; Begg’s test *P* = 0.61). In terms of ACQ-5 scores and FE_NO_, our meta-analysis showed that dupilumab administration significantly decreased the ACQ-5 score and FE_NO_ (SMD = − 4.95, 95% CI: − 7.30 to − 2.60, *P* < 0.001; SMD = − 2.40, 95% CI: − 3.64 to − 1.17, *P* < 0.001, respectively), with high heterogeneity (I^2^ = 100%, *P* < 0.001) (Figs. 4 and 5), but no publication bias (Egger’s test *P* = 0.73; Begg’s test *P* = 0.52; Egger’s test *P* = 0.68; Begg’s test *P* = 0.58, respectively). Similar efficacy was observed in terms of the AM and PM asthma symptom score. Compared with placebo, dupilumab resulted in a greater reduction in the AM and PM asthma symptom scores (SMD = − 5.09, 95% CI: − 6.40 to − 3.77, *P* < 0.001; SMD = − 4.92, 95% CI: − 5.98 to − 3.86, *P* < 0.001, respectively), with high heterogeneity (I^2^ = 99%, *P* < 0.001; I^2^ = 98%, *P* < 0.001, respectively) (Figs. 6 and 7), and no publication bias (Egger’s test *P* = 0.73; Begg’s test *P* = 0.51). Additionally, dupilumab treatment was associated with a significant reduction in severe asthma exacerbation risk (RR = 0.73; 95% CI: 0.67 to 0.79; *P* < 0.001) (Fig. 8), without heterogeneity among studies (I^2^ = 0%, *P* = 0.59), or publication bias (Egger’s test *P* = 0.98; Begg’s test, *P* = 0.62). For AQLQ, our analysis demonstrated that dupilumab treatment significantly increased AQLQ scores (SMD = 4.39, 95% CI: 1.44 to 7.34, *P* = 0.004), with high heterogeneity (I^2^ = 100%, *P* < 0.001) (Fig. 9), and no publication bias (Egger’s test, *P* = 0.27; Begg’s test, *P* = 0.07).

### Safety

**Fig. 10:**
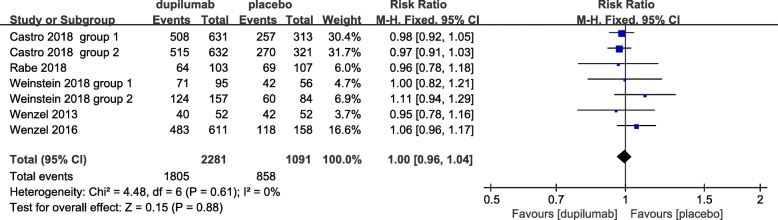
Forest plot of the effect of dupilumab treatment on adverse events verse placebo. Fixed-effects model. RR = relative risk, CI = confidence interval

All studies presented adverse events and dupilumab was relatively tolerated. The overall frequency of adverse events was similar in dupilumab-treated (79.2%) and placebo-treated (78.6%) patients (RR = 1.0; 95% CI: 0.96 to 1.04; *P* = 0.94), with no heterogeneity (I^2^ = 0%, *P* = 0.59) (Fig. 10) and no publication bias (Egger’s test *P* = 1.00; Begg’s test, *P* = 0.69). The incidence of serious adverse events was relatively low in the dupilumab treatment group (2–8.7%). Common adverse events were injection-site reactions, upper respiratory tract infection, headache, nasopharyngitis, bronchitis, and sinusitis (Table 2).

**Table 2:** Summary of adverse events in dupilumab treatment arm

Event	Wenzel 2013 [27]	Wenzel 2016 [15]	Castro 2018 [26]	Rabe 2018 [25]	Weinstein 2018 [24]
Any adverse event	42(80.8)	483(79.1)	1023(81.0)	64 (62.1)	195 (78.3)
Serious adverse event	1 (2.0)	45 (7.4)	104 (8.2)	9 (8.7)	15 (6.0)
Any adverse event leading to death	NM	2(<0.1)	5 (0.4)	0	0
Study discontinuation owing to treatment-emergent adverse event	3 (5.8)	27 (4.4)	63 (5.0)	1 (1.0)	8 (3.0)
Most common adverse events					
Injection-site reactions	15 (28.8)	79 (12.9)	212 (16.8)	9 (8.7)	60 (24.1)
Upper respiratory tract infection	7 (13.5)	216 (35.4)	146 (11.6)	NM	NM
Viral upper respiratory tract infection	3 (5.8)	83 (13.6)	230 (18.2)	9 (8.7)	NM
Headache	6 (11.5)	62 (10.1)	86 (6.8)	NM	NM
Nasopharyngitis	7 (13.5)	59 (9.7)	NM	NM	NM
Bronchitis	NM	51 (8.3)	144 (11.4)	7 (6.8)	NM
Sinusitis	1 (2.0)	36 (5.9)	62 (4.9)	7 (6.8)	NM

Note: Data are n (%)

*Abbreviations*: *NM* not mentioned

### Quality of the individual studies and subgroup analyses

**Fig. 11:**
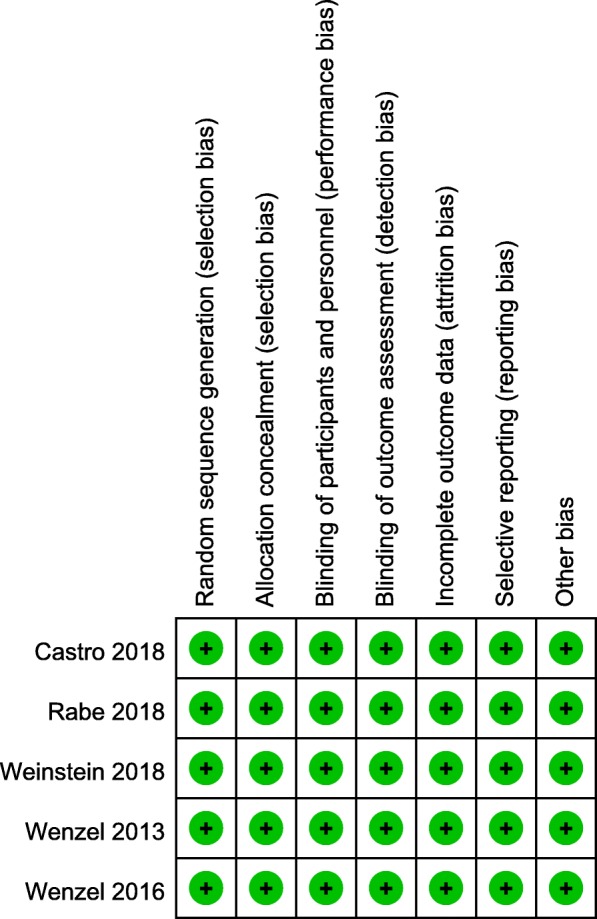
Risk of bias summary of included studies summary

**Fig. 12:**
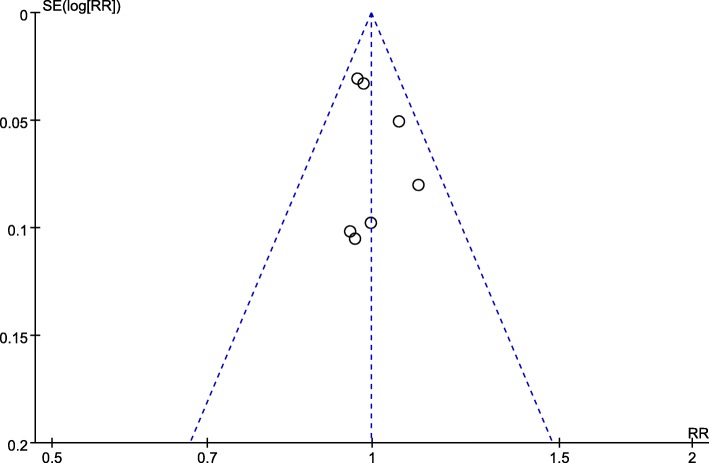
Funnel plot of the included studies evaluated adverse events

All trials had a low risk of bias in terms of the 6 domains (Fig. 11). The publication bias of trials was estimated by visual inspection of the funnel plot and Egger’s test, and there was no publication bias (Fig. 12, *P* = 1.00). According to the funding source, subgroup analyses were performed to assess sponsorship biases. Unfortunately, all studies were funded by a pharmaceutical company (Sanofi or Regeneron Pharmaceuticals). Thus, the results should be expounded with caution. In order to examine the source of heterogeneity, subgroup analyses were conducted for FEV_1_ and ACQ-5 scores. The studies were stratified in terms of the effects model, region of trial, sample size, treatment duration, blood eosinophil count, and sources of funding, but no significant difference was found between groups in terms of FEV_1_ and ACQ-5 scores (Table 3).

**Table 3:** Subgroup analysis and sensitivity analyses of FEV_1_ and ACQ-5 scores in RCTs

Stratification Subgroup analysis	FEV_1_				ACQ-5 scores			
	No. of Patients (trials)	SMD (95% CI)	*P* Value	I_2_, %	No. of Patients (trials)	SMD (95% CI)	*P* Value	I_2_, %
Effects model								
Random-effects model	3218 (7)	4.29 (2.78 to 5.81)	< 0.001	99	2670 (4)	−4.95(−7.30 to −2.60)	< 0.001	100
Fixed effects model	3218 (7)	4.48 (4.32 to 4.62)	< 0.001	99	2670 (4)	−4.24(− 4.40 to − 4.09)	< 0.001	100
Region of study								
The US	104 (1)	4.47 (3.74 to 5.20)	< 0.001	–	104 (1)	−4.53(−5.26 to −3.79)	< 0.001	–
Multinational	3114 (6)	4.31 (2.85 to 5.97)	< 0.001	99	2566 (3)	−5.09(−7.29 to −2.25)	< 0.001	100
No. of subjects								
< 150	651 (4)	3.21 (1.98 to 4.43)	< 0.001	96	104 (1)	−4.53(−5.26 to −3.79)	< 0.001	–
≥150	2567 (3)	5.80 (3.86 to 7.74)	< 0.001	99	2566 (3)	−5.09(−7.29 to −2.25)	< 0.001	100
Treatment duration								
< 50 weeks	1316 (4)	3.35 (2.36 to 4.34)	< 0.001	96	768 (2)	−3.47(−5.48 to −1.46)	< 0.001	96
≥50 weeks	1902 (2)	6.74 (6.25 to 7.23)	< 0.001	77	1902 (2)	−6.39(−9.89 to −2.89)	< 0.001	100
Blood eosinophil count								
< 300 eosinophils per μl	1536 (5)	5.56 (2.59 to 8.53)	< 0.001	99	388 (1)	−1.80(−2.08 to − 1.52)	< 0.001	–
≥300 eosinophils per μl	1269 (4)	2.05 (0.97 to 3.12)	< 0.001	98	380 (2)	−2.43(−6.52 to − 1.66)	< 0.001	99
Sources of funding								
Pharmaceutical company		4.29 (2.78 to 5.81)	< 0.001	99		−4.95(−7.30 to −2.60)	< 0.001	100
Non-pharmaceutical company	0 (0)	–	–	–	–	–	–	–

*Abbreviations*: *FEV1* forced expiratory volume in 1 s, *ACQ-5* 5-item Asthma Control Questionnaire

## Discussion

To our knowledge, this is the first meta-analysis and comparison of all published data of RCTs on the use of dupilumab for the treatment of uncontrolled asthma. Our study indicated that dupilumab treatment was relatively tolerated and could significantly improve FEV_1_ and quality of life, and reduced the disease symptom score and severe exacerbations risk in patients with uncontrolled asthma.

Asthma is a heterogeneous disease [1]. Previous studies have indicated that the Th2 cytokines IL-4 and IL-13 play a part in asthma [28, 29]. Dupilumab is an IL-4R antagonist that inhibits the IL-4 and IL-13 signaling pathway. Several clinical trials have been performed evaluating the effect of dupilumab in uncontrolled asthma. However, the evidence was insufficient to allow sound conclusions to be drawn, because the sample sizes varied; therefore, further analyses were necessary.

Based on pooled analyses, we found that dupilumab could significantly improve FEV_1_ in patients with uncontrolled asthma. The clinical relevance of this finding may be clinically important, suggesting a potential effect of dupilumab on airway remodeling. However, a previous study [24] failed to show a significant effect on lung function as compared with placebo, which may be attributed to the small sample size and unselected population of patients with asthma. Compared to the previous study, we identified a selected population of patients with uncontrolled asthma. Moreover, we performed TSA to estimate the required information size for analysis, which can decrease the risk of random errors. Thus, our results may be more plausible. In addition, the different outcomes between our study and the previous trial suggested that dupilumab may be valid only in a targeted subgroup with uncontrolled asthma.

Three trails [15, 26, 27] showed that dupilumab could significantly reduce ACQ-5 scores and asthma symptom scores, which was consistent with our findings. AQLQ is a disease-specific health-related quality of life instrument that contains 32 items has been proven to be responsive in before–after studies and in clinical trials [30]. In our study, we used the AQLQ score to assess the quality of life of patients. Dupilumab treatment significantly improved AQLQ score in patients with uncontrolled asthma, this result was consistent with those of previous reviews. However, the mean change in AQLQ was less than the minimally important clinical difference of 0.5 units [31]. Thus, the relation of this finding to patients may not be clinically significant.

Given that nitric oxide (NO)-synthase activity and NO production are promoted by IL-13, FE_NO_ levels can be used as a biomarker of Th2 inflammation and of IL-13 levels in the bronchial mucosa [32]. Therefore, FE_NO_ levels could decrease after treatment with anti-IL-4/13 therapy [33]. The current meta-analysis further confirmed this theory: our study showed a significantly decrease in FE_NO_ levels after dupilumab treatment. Similarly, all previous trials of dupilumab treating uncontrolled asthma observed a reduction in the FE_NO_ level, which is consistent with the mechanism of action of dupilumab.

Severe asthma exacerbations are related to substantial mortality [34]. Reducing asthma exacerbation risk is a key target of disease management. The part of Th2 inflammation is about 50% of asthmatic patients, in whom proinflammatory cytokines abnormally product, such as IL-4, IL-5, and IL-13, which induce IgE synthesis and eosinophilic inflammation [35]. Trials using specific inhibitors have indicated a relation between eosinophils and the pathogenesis of asthma exacerbations [36, 37]. Therefore, it is hypothesized that anti-IL-4 and anti-IL-13 therapies could decrease asthma exacerbation by inhibiting eosinophilic and airway inflammation. In further sustain of this concept, our study showed a significant reduction in severe exacerbation rates with dupilumab treatment. The improvements in asthma-related quality of life with dupilumab treatment may contribute to preventing severe asthma exacerbations.

The whole safety profile of dupilumab was equivalent to that of placebo in all RCTs, and the common adverse events were injection-site reactions, upper respiratory tract infection, headache, nasopharyngitis, bronchitis, and sinusitis. Four trials [15, 25–27] showed that the rates of upper respiratory tract infection in the dupilumab-treated group were low and similar to placebo group. However, all RCTs included revealed that the incidence of injection-site reactions was slightly higher in the dupilumab regimens. Therefore, more data are required to support the safety profile of dupilumab in terms of injection-site reactions.

This meta-analysis has some shortcomings. First, the severity of uncontrolled asthma and baseline therapy varied among studies; thus, it is impossible to probe the impact of these factors on results. Second, significant heterogeneity existed among trials evaluating FEV1, ACQ-5 score, FE_NO_, and AQLQ, possible sources of heterogeneity might include different geographic regions and races. Third, we combined 2 or 3 intervention groups into a single group according to the Cochrane handbook, regardless of differences in intervention dosage, which made it hard to determine the optimal dose. Last, as all the RCTs involved the pharmaceutical industry, the positive outcomes should be expounded cautiously. Our study also had some strengths; for instance, our patient selection was highly consistent, and all trials were of high quality, such that the internal validity of our meta-analysis was high.

## Conclusions

The current meta-analysis demonstrated that dupilumab treatment was relatively tolerated and significantly improved FEV_1_, symptoms, asthma control, and quality of life, and reduced severe exacerbation risk in patients with uncontrolled asthma. Thus, dupilumab may be effective and safe for the treatment of uncontrolled asthma. These results highlight the significance of selecting the population patient of asthma that could obtain clinical benefit from dupilumab. Injection-site reaction was the most frequently reported adverse event in all studies involving dupilumab treatment. Further long-term trials are required to determine the optimum dose of dupilumab for asthma.

## Abbreviations

ACQ-5: 5-item Asthma Control Questionnaire
AQLQ: The Asthma Quality of Life Questionnaire
FE_NO_: Fractional exhaled nitric oxide
FEV_1_: Forced expiratory volume in 1 s
IgE: Immunoglobulin E
IL: Interleukin
NM: Not mentioned
NO: Nitric oxide
PEF: Peak expiratory flow
q1w: Once weekly
q2w: Every 2 weeks
q4w: Every 4 weeks
RCTs: Randomized, placebo-controlled studies
RR: Relative risk
SMD: Standard mean differences
Th2: T-helper-2-cell

## References

[1] Disease GBD, Injury I, Prevalence C (2017). Global, regional, and national incidence, prevalence, and years lived with disability for 328 diseases and injuries for 195 countries, 1990-2016: a systematic analysis for the global burden of Disease study 2016. Lancet.

[2] Peters SP, Ferguson G, Deniz Y, Reisner C (2006). Uncontrolled asthma: a review of the prevalence, disease burden and options for treatment. Respir Med.

[3] Kerkhof M, Tran TN, Soriano JB, Golam S, Gibson D, Hillyer EV, Price DB (2018). Healthcare resource use and costs of severe, uncontrolled eosinophilic asthma in the UK general population. Thorax.

[4] Lange P, Parner J, Vestbo J, Schnohr P, Jensen G (1998). A 15-year follow-up study of ventilatory function in adults with asthma. N Engl J Med.

[5] Chung KF, Wenzel SE, Brozek JL, Bush A, Castro M, Sterk PJ, Adcock IM, Bateman ED, Bel EH, Bleecker ER (2014). International ERS/ATS guidelines on definition, evaluation and treatment of severe asthma. Eur Respir J.

[6] Wenzel S, Wilbraham D, Fuller R, Getz EB, Longphre M (2007). Effect of an interleukin-4 variant on late phase asthmatic response to allergen challenge in asthmatic patients: results of two phase 2a studies. Lancet.

[7] Locksley RM (2010). Asthma and allergic inflammation. Cell.

[8] Woodruff PG, Modrek B, Choy DF, Jia G, Abbas AR, Ellwanger A, Koth LL, Arron JR, Fahy JV (2009). T-helper type 2-driven inflammation defines major subphenotypes of asthma. Am J Respir Crit Care Med.

[9] Corren J, Lemanske RF, Hanania NA, Korenblat PE, Parsey MV, Arron JR, Harris JM, Scheerens H, Wu LC, Su Z (2011). Lebrikizumab treatment in adults with asthma. N Engl J Med.

[10] Chung KF (2015). Targeting the interleukin pathway in the treatment of asthma. Lancet.

[11] Gandhi NA, Pirozzi G, Graham NMH (2017). Commonality of the IL-4/IL-13 pathway in atopic diseases. Expert Rev Clin Immunol.

[12] Shirley M (2017). Dupilumab: First Global Approval. Drugs.

[13] Global Initiative for Asthma (GINA). 2009 Global strategy for asthma management and prevention. https://ginasthma.org/. Accessed 22 June 2018.

[14] Global Initiative for Asthma (GINA). 2015 Global strategy for asthma management and prevention. https://ginasthma.org/wp-content/uploads/2016/01/GINA_Report. Accessed 22 June 2018.

[15] Wenzel S, Castro M, Corren J, Maspero J, Wang L, Zhang B, Pirozzi G, Sutherland ER, Evans RR, Joish VN (2016). Dupilumab efficacy and safety in adults with uncontrolled persistent asthma despite use of medium-to-high-dose inhaled corticosteroids plus a long-acting beta<inf>2</inf> agonist: a randomised double-blind placebo-controlled pivotal phase 2b dose-ranging trial. Lancet.

[16] Juniper EF, Svensson K, Mork AC, Stahl E (2005). Measurement properties and interpretation of three shortened versions of the asthma control questionnaire. Respir Med.

[17] Busse WW, Maspero JF, Rabe KF, Papi A, Wenzel SE, Ford LB, Pavord ID, Zhang B, Staudinger H, Pirozzi G (2018). Liberty asthma QUEST: phase 3 randomized, double-blind, placebo-controlled, parallel-group study to evaluate Dupilumab efficacy/safety in patients with uncontrolled, moderate-to-severe asthma. Adv Ther.

[18] Moher D, Liberati A, Tetzlaff J, Altman DG, Group P (2009). Preferred reporting items for systematic reviews and meta-analyses: the PRISMA statement. J Clin Epidemiol.

[19] Higgins J. Green S. Cochrane handbook for systematic reviews of interventions version 5.1. 0. The Cochrane Collaboration, 2011. 2013.

[20] Higgins JP, Thompson SG (2002). Quantifying heterogeneity in a meta-analysis. Stat Med.

[21] Egger M, Davey Smith G, Schneider M, Minder C (1997). Bias in meta-analysis detected by a simple, graphical test. BMJ.

[22] Brok J, Thorlund K, Wetterslev J, Gluud C (2009). Apparently conclusive meta-analyses may be inconclusive—trial sequential analysis adjustment of random error risk due to repetitive testing of accumulating data in apparently conclusive neonatal meta-analyses. Int J Epidemiol.

[23] Thorlund K, Engstrøm J, Wetterslev J, Brok J, Imberger G, Gluud C (2011). User manual for trial sequential analysis (TSA).

[24] Weinstein SF, Katial R, Jayawardena S, Pirozzi G, Staudinger H, Eckert L, Joish VN, Amin N, Maroni J, Rowe P (2018). Efficacy and safety of dupilumab in perennial allergic rhinitis and comorbid asthma. J Allergy Clin Immunol.

[25] Rabe KF, Nair P, Brusselle G, Maspero JF, Castro M, Sher L, Zhu H, Hamilton JD, Swanson BN, Khan A (2018). Efficacy and safety of Dupilumab in glucocorticoid-dependent severe asthma. N Engl J Med.

[26] Castro M, Corren J, Pavord ID, Maspero J, Wenzel S, Rabe KF, Busse WW, Ford L, Sher L, FitzGerald JM (2018). Dupilumab efficacy and safety in moderate-to-severe uncontrolled asthma. N Engl J Med.

[27] Wenzel S, Ford L, Pearlman D, Spector S, Sher L, Skobieranda F, Wang L, Kirkesseli S, Rocklin R, Bock B (2013). Dupilumab in persistent asthma with elevated eosinophil levels. New England J Med.

[28] Kay AB (2006). The role of T lymphocytes in asthma. Chem Immunol Allergy.

[29] Gandhi NA, Bennett BL, Graham NM, Pirozzi G, Stahl N, Yancopoulos GD (2016). Targeting key proximal drivers of type 2 inflammation in disease. Nat Rev Drug Discov.

[30] Juniper EF, Guyatt G, Epstein R, Ferrie P, Jaeschke R, Hiller TK (1992). Evaluation of impairment of health related quality of life in asthma: development of a questionnaire for use in clinical trials. Thorax.

[31] Juniper EF, Guyatt GH, Willan A, Griffith LE (1994). Determining a minimal important change in a disease-specific quality of life questionnaire. J Clin Epidemiol.

[32] Barranco P, Phillips-Angles E, Dominguez-Ortega J, Quirce S (2017). Dupilumab in the management of moderate-to-severe asthma: the data so far. Ther Clin Risk Manag.

[33] Chibana K, Trudeau JB, Mustovich AT, Hu H, Zhao J, Balzar S, Chu HW, Wenzel SE (2008). IL-13 induced increases in nitrite levels are primarily driven by increases in inducible nitric oxide synthase as compared with effects on arginases in human primary bronchial epithelial cells. Clin Exp Allergy.

[34] Masoli M, Fabian D, Holt S, Beasley R, Global initiative for asthma P (2004). The global burden of asthma: executive summary of the GINA dissemination committee report. Allergy.

[35] Bagnasco D, Ferrando M, Varricchi G, Passalacqua G, Canonica GW (2016). A critical evaluation of anti-IL-13 and anti-IL-4 strategies in severe asthma. Int Arch Allergy Immunol.

[36] Green RH, Brightling CE, McKenna S, Hargadon B, Parker D, Bradding P, Wardlaw AJ, Pavord ID (2002). Asthma exacerbations and sputum eosinophil counts: a randomised controlled trial. Lancet.

[37] Nadif R, Siroux V, Oryszczyn MP, Ravault C, Pison C, Pin I, Kauffmann F, Epidemiological study on the G, environment of A (2009). Heterogeneity of asthma according to blood inflammatory patterns. Thorax.

